# HPLC Enantioseparation of Rigid Chiral Probes with Central, Axial, Helical, and Planar Stereogenicity on an Amylose (3,5-Dimethylphenylcarbamate) Chiral Stationary Phase

**DOI:** 10.3390/molecules27238527

**Published:** 2022-12-03

**Authors:** Simona Rizzo, Tiziana Benincori, Francesca Fontana, Dario Pasini, Roberto Cirilli

**Affiliations:** 1 CNR Istituto di Scienze e Tecnologie Chimiche “Giulio Natta”, Via C. Golgi 19, 20133 Milano, Italy; 2 Dipartimento di Scienza e Alta Tecnologia, Università degli Studi dell’Insubria, Via Valleggio 11, 22100 Como, Italy; 3 Dipartimento di Ingegneria e Scienze Applicate, Università di Bergamo, Viale Marconi 5, 24044 Dalmine, Italy; 4 CSGI Bergamo R.U., Viale Marconi 5, 24044 Dalmine, Italy; 5 Department of Chemistry and INSTM Research Unit, University of Pavia, 27100 Pavia, Italy; 6 Centro Nazionale per il Controllo e la Valutazione dei Farmaci, Istituto Superiore di Sanità, Viale Regina Elena 299, 00161 Rome, Italy

**Keywords:** planar stereogenicity, axial stereogenicity, trypticene, helicene, HPLC enantioseparation, amylose (3,5-dimethylphenylcarbamate), Chiralpak AD-3

## Abstract

The chiral resolving ability of the commercially available amylose (3,5-dimethylphenylcarbamate)-based chiral stationary phase (CSP) toward four chiral probes representative of four kinds of stereogenicity (central, axial, helical, and planar) was investigated. Besides chirality, the evident structural feature of selectands is an extremely limited conformational freedom. The chiral rigid analytes were analyzed by using pure short alcohols as mobile phases at different column temperatures. The enantioselectivity was found to be suitable for all compounds investigated. This evidence confirms that the use of the amylose-based CSP in HPLC is an effective strategy for obtaining the resolution of chiral compounds containing any kind of stereogenic element. In addition, the experimental retention and enantioselectivity behavior, as well as the established enantiomer elution order of the investigated chiral analytes, may be used as key information to track essential details on the enantiorecognition mechanism of the amylose-based chiral stationary phase.

## 1. Introduction

The polysaccharide-based chiral stationary phases (CSPs) are now routinely used in ultra-high-performance liquid chromatography (UHPLC) [1,2,3], high-performance liquid chromatography (HPLC) [4], as well as supercritical fluid chromatography (SFC) [5] enantiomeric separations. Their application allows the resolution of a broad range of chiral compounds from nano to preparative scale under multimodal elution conditions.

Despite their success, at present, there is no reliable way to predict whether or not an enantioselective separation will be achieved on a given polysaccharide-based CSP. Although in silico molecular models capable of mimicking the behavior of the polysaccharide selectors have been developed and, in some circumstances, successfully applied to actual enantioseparations [6], the research of enantioselective conditions is still carried out through the evaluation of literature data or the screening of commercially available columns. The importance of predictive tools in the enantioselective HPLC analysis of chiral compounds has attracted interest from many researchers and stimulated the study of the interactions involved in the enantiorecognition process promoted by polysaccharide derivatives through NMR, IR, HPLC, and computational techniques [7,8,9,10,11]. 

One strategy for the development of new pieces of knowledge on the mechanistic aspect of the enantioseparation process is the design of tailored chiral probes that display a chromatographic behavior traceable to specific structural elements [12,13,14,15,16,17,18,19,20]. This approach allows the construction of reliable structure–enantioselectivity relationships and, indirectly, the identification of the key portions of the selector involved in the enantioseparation. 

**Figure 1:**
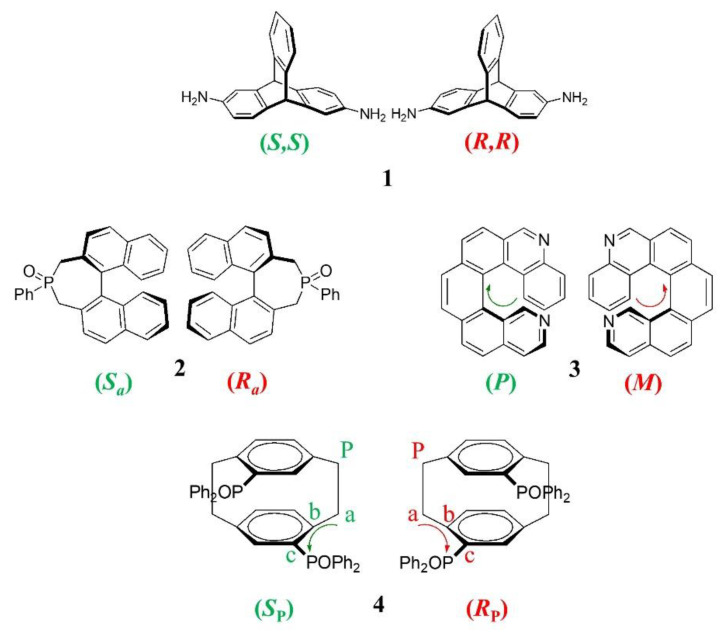
Chemical structures and stereochemical descriptors of enantiomers of chiral compounds **1**–**4** representative of the four stereogenic elements: **1** central stereogenicity, **2** axial stereogenicity, **3** helical stereogenicity, **4** planar stereogenicity.

In this context, this work reports on the enantioseparation of a small set of chiral compounds (compounds **1**–**4**, Figure 1) on the commercially available amylose (3,5-dimethylphenylcarbamate) (ADMPC)-based Chiralpak AD-3 CSP. The amylose derivative is considered to be one of the most effective selectors for achieving chiral resolution, and it is used in the preparation of commercially available chiral packing materials [4,10]. As shown in Figure 1, for each kind of stereogenicity (central, axial, helical, and planar), a representative chiral analyte was chosen. 

The triptycene **1** has a paddle-wheel-shaped structure consisting of three benzene rings fused to a bicyclo [2.2.2]octatriene bridgehead system. The unique three-dimensional rigid structure of **1** and the ample possibilities for the installment of reactive positions are attractive points for this class of molecules in order to either modify/tune the π-scaffold and introduce reactive handles for further manipulation and integration into nanostructures. Compared to other *C_2_*-symmetrical chiral synthons, such as trans-1,2-disubstituted cycloalkanes and 1,1,2,2-tetrasubstituted ethane-based scaffolds, triptycenes exhibit outstanding features that are attractive for the development of new functional molecular design, including a robust chiral backbone and extremely limited conformational structure. Recent advances in the synthesis of chiral triptycenes and in their introduction as molecular scaffolds for the assembly of functional supramolecular materials have been recently reviewed [21].

Compound **2** is prepared by oxidation of the enantiomers of the corresponding phosphane, the 4-phenyl-4,5- dihydro-3H-dinaphtho [2,1-c;10,20-e]phosphepine (Binepine), which is a versatile monodentate ligand of transition metals, Rh in particular, employed as a mediator in a wide variety of successful homogeneous stereoselective reactions. The 2,7-dihydrophosphepine oxide **2** does not display any catalytic activity. It can be prepared by oxidation of the enantiomers of Binepine and employed as an intermediate for the synthesis of 3,5-dialkyl-Binepines [22]. 

The diaza [6]helicene (compound **3**) is characterized by an extensively conjugated, inherently chiral structure capable of fluorescence and phosphorescence emission and endowed with interesting optical properties [23]; besides, the presence of the nitrogen atoms allows functionalization, quaternarization, or complexation with metal ions [24], thus opening the way to the preparation of active materials for chiral sensing and optoelectronics [25,26,27]. 

The planar chiral diphosphane oxide with a *p*-cyclophane scaffold (compound **4**) is the key compound in the resolution process of phanephos, a very popular *C_2_*-symmetric diphosphane, employed as a ligand of Ru(II)- and Rh(I) in asymmetric hydrogenation of stereogenic C=O [28] and C=C double bonds [29].

Although compounds **1**–**4** are profoundly different from a structural point of view, they share a specific characteristic, namely that of being rigid and bulky molecules with extremely limited conformational freedom because their structure is fixed by a ring system (compounds **1**, **2,** and **4**) or formed by an extended aromatic system with non-coplanar extremities (compound **3**). One challenge in selector–selectand docking is the treatment of molecular flexibility and changes in the conformational states. Any change in the selectand conformation can lead to a large difference in the resulting docked poses. Thus, the use of rigid chiral molecules such as **1**–**4** is expected to be an intriguing strategy to facilitate enantiorecognition process investigations by docking studies. 

To support this hitherto untapped application and to verify the versatility of the Chiralpak AD-3 CSP, this work aimed to investigate the chromatographic behavior of **1**–**4** on the amylose-type CSP by (i) using different polar organic eluents, (ii) changing the column temperature, and (iii) determining the elution order of enantiomers in all conditions investigated.

## 2. Results and Discussion

### 2.1. HPLC Enantioseparation under Polar Organic Mode

Before discussing the chromatographic results on the enantioseparation of **1**–**4**, it is useful to remember the stereogenic elements of such unusual chiral molecules. The chirality of triptycene **1** is due to the NH_2_ substituents at the 2,6-positions that make two bridgehead carbon atoms as stereogenic centers. The 2,7-dihydrophosphepine **2** is characterized by a 1,1′-binaphthalene scaffold and, consequently, displays an atropoisomeric framework as the stereogenic element. The third chiral compound studied in this work is an inherently chiral diaza [6]helicene (compound **3**) with a nonplanar screw-shaped structure formed from fused benzene and pyridine rings. The last term of the series is a planar chiral diphosphane oxide with a *p*-cyclophane scaffold (compound **4**). According to the CIP rules concerning the attribution of the configurational descriptors, (*S*_P_) and (*R*_P_), to stereogenic planar molecules, (i) the stereogenic plane of **4** is indifferently one of the two planes containing the aromatic ring, two phosphorous atoms, and two methylene groups; (ii) the first priority atom located out of the plan (indicated as P in Figure 1) is indifferently one of the two equivalent methylene carbons of the cyclophane bridge; (iii) from the pilot atom P, starting from the atom directly connected to it (indicated as *a* in Figure 1), the sequence of atoms is that along with the *b* and *c* atoms, which have the highest CIP priority; (iv) the sequence is clockwise for the (*R*_P_)-enantiomer and counterclockwise for the (*S*_P_)-enantiomer. 

Compounds **1**–**4** are, in all cases, enantiomerically stable at room temperature and thus resolvable. 

**Figure 2:**
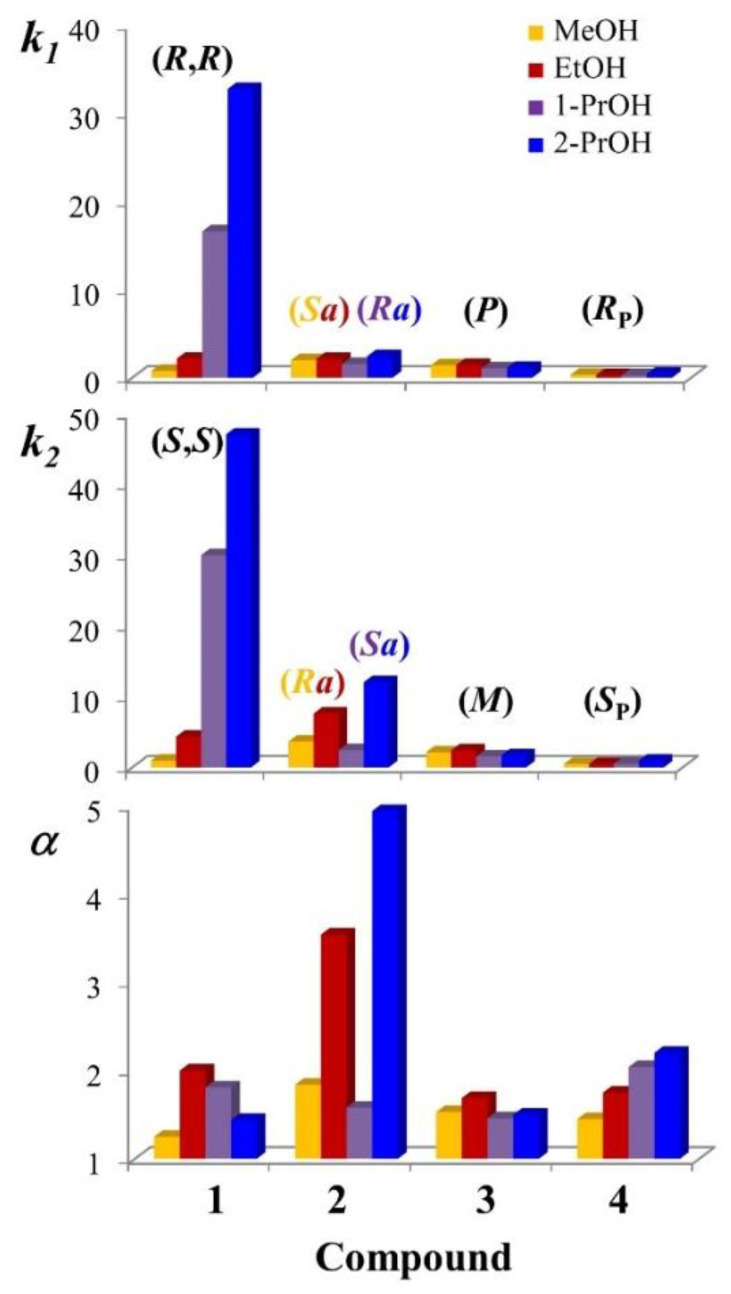
Effect of mobile phase on the retention (*k_1_* and *k_2_*) and enantioseparation (*α*) factors of **1**–**4**. Chromatographic conditions: column, Chiralpak AD-3 (100 mm × 4.6 mm, 3 μm); flow rate, 1 mL/min; temperature, 25 °C; detection, UV and CD at 241 nm. 2-PrOH: 2-propanol; 1-PrOH: 1-propanol, EtOH: ethanol; MeOH: methanol.

Figure 2 and Table S1 of Supplementary Materials resume the retention (*k*) and enantioseparation (*α*) factors obtained by: (i) setting the column temperature at 25 °C, (ii) using the 100 mm × 4.6 mm Chiralpak AD-3 column packed with 3-μm ADMPC-based particles, and (iii) selecting neat methanol, ethanol, 1-propanol, and 2-propanol as mobile phases. 

**Figure 3:**
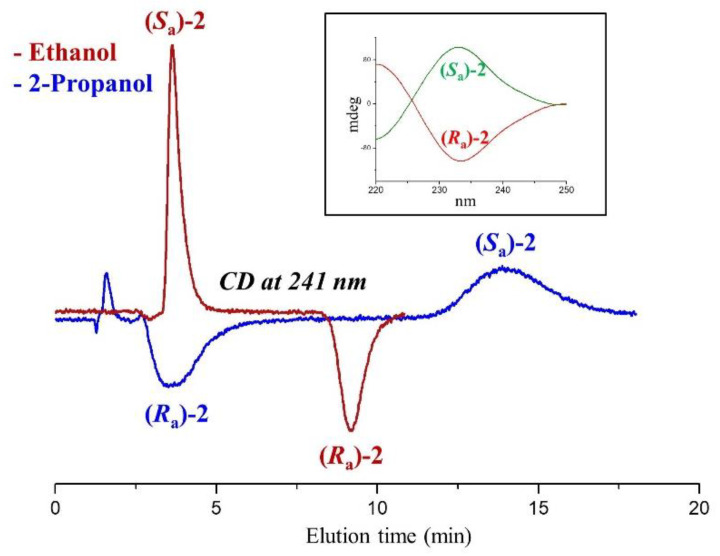
Typical chromatograms illustrating the differences in enantiomer elution order of **2** using pure ethanol and 2-propanol as mobile phases. Chromatographic conditions: column, Chiralpak AD-3 (100 mm × 4.6 mm, 3 μm); flow rate, 1 mL/min; temperature, 25 °C; detection, CD at 241 nm. 2-PrOH: 2-propanol; 1-PrOH: 1-propanol, EtOH: ethanol; MeOH: methanol. Inset: CD spectra of enantiomers of **2**.

The most marked dependence of enantioselectivity from mobile phase composition was recorded for compound **2**. As the less retained enantiomer elutes at close retention times, this variability is attributed essentially to the different retention of the more retained enantiomer. The maximum value of the enantioseparation factor was recorded in 2-propanol (α = 4.94) and the minimum in 1-propanol (α = 1.58). In both elution modes, the enantiomer (*R_a_*)-**2** was eluted before (*S_a_*)-**2**. Passing to methanol and ethanol, the enantiomer elution reversed, and the enantioselectivity became almost double. As visible in Figure 3, the evaluation of the sign of the ellipticity recorded during chromatography leads to a solid interpretation of the elution order of enantiomers.

The (*Sa*)-**2** enantiomer was eluted as the first species from the Chiralpak AD-3 column and showed a positive circular dichroism (CD) peak at 241 nm. The offline CD signal assumed the same positive sign at the wavelength of 241 nm (see the CD spectrum depicted in Figure 3). Passing from ethanol to 2-propanol, the sign of the online CD peaks reversed, and the (*Ra*)-**2** enantiomer became the less retained species.

The extreme variability of enantioselectivity and the inversion of enantiomer elution order can be attributed to the ability of the molecules of alcohol used in the mobile phase to alter the conformation of the polymeric ADMPC selector as well as the size and shape of the chiral periodical helical grooves occurring along the polymeric backbone [10,29,30]. The incorporation of molecules of alcohol within the nano-sized chiral cavities in the ADMPC structure leads to a fine modulation of their steric environment. Thus, depending on the type of molecules incorporated, the wetted chiral cavities can differently select the portions of the enantiomers of chiral analyte **2** that can interact with the carbamate sites of selector through hydrogen bond and dipole-dipole interactions [12,29,30].

Contrary to what was observed in the case of compound **2**, the enantiomeric separation of **3** was weakly influenced by the nature of alcoholic eluent. Notably, the retention factor of the more retained enantiomer was unusually low in the polar organic conditions used and ranged from 1.57 (with 1-propanol) to 2.43 (with ethanol).

For compound **4**, the enantioselectivity increased in parallel to the chain-lengthening from methanol to 1-propanol. A further increase in the enantioseparation factor was recorded using 2-propanol. Under these eluent conditions, the enantioseparation factor values changed from 1.45 to 2.2. As in the case of **3**, the retention of both enantiomers was very weak, and the retention factor values were lower than 1, irrespective of the nature of the mobile phase employed. The poor retentive properties of the selector can be attributed to the strong competitive interactions established by alcohol molecules with the active sites of the Chiralpak AD-3 CSP. This evidence leads to the hypothesis that P=O groups of **4** are involved in hydrogen bonds with the carbamate groups of the amylose derivative [10], and this type of interaction is the driving force of the enantiorecognition process.

To conclude this section, let us look at the chromatographic data obtained for compound **1**. 

Both the retention factor values for the first and second eluted enantiomers dramatically and progressively increased using the following series of solvents: methanol < ethanol < 1-propanol < 2-propanol. Using 2-propanol, the enantioseparation factor of the second eluted (*R*,*R*)-enantiomer reached the remarkable value of 47.11. For the same enantiomer, the value collapsed to 0.97 with methanol. Despite this trend, the highest enantioselectivity was observed in ethanol (α = 1.58), while using 2-propanol, the enantioseparation factor was only 1.44. A possible explanation for interpreting the remarkable retention of **1** employing 2-propanol as a mobile phase is the involvement of the NH_2_ group in one or more strong and poorly selective hydrogen bonds with the carbamate moieties of the (ADMPC)-based CSP.

### 2.2. Thermodynamic Aspects of Enantioseparation

The thermodynamic parameters associated with the enantioseparation of compounds **1**–**4** on the Chiralpak AD-3 CSP were determined by correlating the enantioseparations factors recorded between 25 and 45 °C and the column temperature. According to the following Equation:

ln α = −∆∆H°/RT + ∆∆S°/R(1)

the differences between the two enantiomers in enthalpy (∆∆H°) and entropy (∆∆S°) of adsorption onto stationary phase were calculated from the slope and intercept, respectively, of ln α vs. 1/T plots (van′t Hoff plots). 

Table 1 shows the enantioseparation factors recorded at 25 °C and the thermodynamic parameters obtained by van’t Hoff analysis.

It follows from Equation (1) that, for the chiral separations in which both the terms ∆∆H° and ∆∆S° were characterized by equal signs (positive or negative), it was possible to calculate the isoenantioselective temperature, namely the temperature at which enthalpy-entropy compensation occurs (|T_ISO_∆∆S°| = | ∆∆H°|) and the enantiomers coelute (α = 1). 

**Figure 4:**
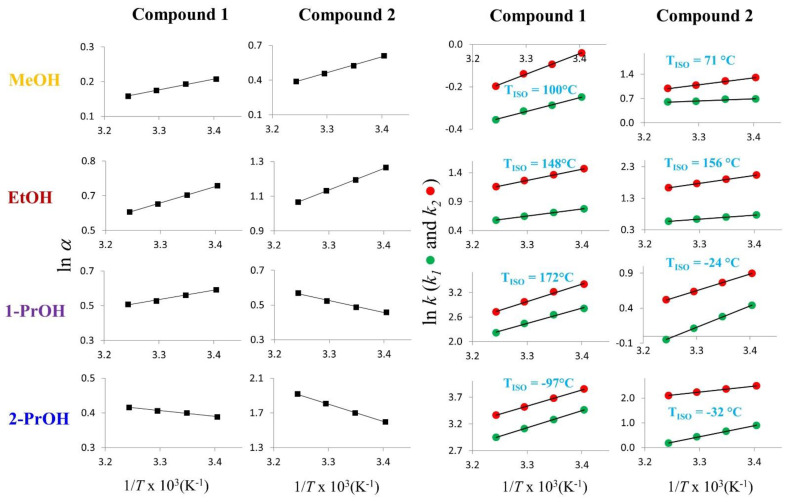
Plots of ln *k* vs. 1/*T* ×10^3^ and ln *α* vs. 1/*T* ×10^3^ for **1** and **2**. Chromatographic conditions: column, Chiralpak AD-3 (100 mm × 4.6 mm, 3 μm); flow rate, 1 mL/min; temperature, 25 °C; detection, CD at 241 nm. 2-PrOH: 2-propanol; 1-PrOH: 1-propanol, EtOH: ethanol; MeOH: methanol.

An inspection of the T_ISO_ values shown in Table 1 reveals that when the column temperatures were higher than the computed T_ISO_ (entries 4, 7, 8, 14, 15, 16), ∆∆H° and ∆∆S° assumed positive signs, and the enantioseparation process occurred within the entropy-controlled domain (|T∆∆S°| > | ∆∆H°|). Accordingly, the separation factors recorded at 45 °C were higher than those recorded at 25 °C. As an example, the separation factor of **2** in the presence of 2-propanol changed from 4.94 to 6.82 as a result of increased temperature. Vice versa, when the column temperatures were lower than the T_ISO_ values, the enantiodiscrimination was enthalpy-driven (|T∆∆S°| < | ∆∆H°|), and the separation factors lowered as temperature increased. In no case, by increasing the temperature in the range of values explored, a change in enantiomer elution order was observed. As representative examples of enantioseparations occurring within entropy and enthalpy domains, Figure 4 shows the van’t Hoff plots obtained by variable-temperature analysis of **1** and **2**.

It is interesting to note that by changing the eluent conditions from pure methanol and ethanol (entries 5 and 6 of Table 1 and Figure 4) to 1-propanol and 2-propanol (entries 7 and 8 of Table 1 and Figure 4), T_ISO_ of **2** becomes lower than room temperature and the elution order of the enantiomers reverses. This once again proves that the temperature of isoelution is a critical parameter to be considered for controlling enantioseparation. Finally, although the enantioseparation of **1** was entropy-controlled with 2-propanol and enthalpy-driven in the other elution conditions, the enantiomer elution order was the same with the (*R*,*R*)-eluted before than (*S*,*S*)-enantiomer.

## 3. Materials and Methods

### 3.1. Reagents and Chemicals

HPLC-grade solvents were obtained from Aldrich (Milan, Italy) and filtered (0.22 μm filter) before use. HPLC analyses were carried out on a Chiralpak AD-3 (100 mm × 4.6 mm, 3 μm) column (Chiral Technologies Europe, Illkirch-Graffenstaden, France). 

Compounds **1**-**4** were synthesized according to previously reported procedures [31,32,33,34,35].

### 3.2. Instruments

HPLC apparatus consisted of a Perkin-Elmer (Norwalk, CT, USA) 200 LC pump equipped with a Rheodyne (Cotati, CA, USA) injector, a 50-μL sample loop, an HPLC Perkin- Elmer oven, and a Jasco (Jasco, Tokyo, Japan) Model CD 2095Plus UV/CD detector. The signal was acquired and processed by Clarity software (DataApex, Prague, Czech Republic). 

The CD spectra of the enantiomers of **2** were recorded at 25 °C by using a Jasco Model J-700 spectropolarimeter. The optical path was 1 mm. The spectra are averagely computed over four instrumental scans, and the intensities are presented in terms of ellipticity values (mdeg).

The absolute configuration of the enantiomers was determined in previous works [30,31,32,33].

### 3.3. HPLC Operating Conditions

Fresh standard solutions of chiral samples were prepared by dissolving the analytes in ethanol solution (concentration about 0.5 mg mL^−1^). The injection volume was 10–30 μL. Solvents and samples were filtered through 0.22-μm filters. The flow rate was set at 1.0 mL min^−1^. The hold-up time was estimated by using 1,3,5-tri-tert-butylbenzene as a marker and pure ethanol as a mobile phase.

## 4. Conclusions

The ADMPC-based Chiralpak AD-3 CSP has been tested in HPLC enantioseparation of four chiral compounds, each of which represents a kind of stereogenicity. The outcomes of the enantioselective HPLC analysis carried out in polar organic conditions indicate that Chiralpak AD-3 CSP can discriminate the enantiomers of all compounds investigated, regardless of their rigid stereogenic element, but its performance is significantly influenced by column temperature and nature of the polar organic mobile phase, which impact the conformation of the polymeric ADMPC selector and the size and shape of its chiral cavities. In particular, in the case of compound **2**, an inversion of the enantiomer elution order occurred, passing from methanol and ethanol to 1-propanol and 2-propanol. Thus, besides proposing an effective approach to the HPLC isolation of enantiopure forms of **1**–**4**, this work adds new information on the resolving capability of the amylose-based Chiralpak AD-3 CSP and highlights the importance of considering mobile phase composition and temperature as key parameters to effectively establish the chiral recognition mechanism of this versatile chromatographic packing material. Finally, tuning molecular rigidity can be an effective strategy for controlling and studying the interactions of the chiral analytes with the active sites of the polysaccharide-based CSPs.

## Figures and Tables

**Table 1:** Absolute configuration (AC) of the first eluted enantiomer, enantioseparation factors at 25 °C, and thermodynamic data of **1**–**4.** Chromatographic conditions: column, Chiralpak AD-3 (100 mm × 4.6 mm, 3 μm); flow rate, 1 mL/min; temperature, 25 °C; detection, CD at 241 nm. 2-PrOH: 2-propanol; 1-PrOH: 1-propanol, EtOH: ethanol; MeOH: methanol. NA: not applicable.

Entry	Compound	Mobile Phase	AC First Eluted/CD Sign at 241 nm	α (25 °C)	∆∆H° (kcal/mol)	∆∆S° (e.u.)	T_ISO_ (°C)
1	**1**	MeOH	(*R*,*R*)/(−)	1.23	−0.62	−1.65	100
2		EtOH	(*R*,*R*)/(−)	2.00	−1.40	−3.32	148
3		1-PrOH	(*R*,*R*)/(−)	1.81	−1.06	−2.38	172
4		2-PrOH	(*R*,*R*)/(−)	1.47	0.33	1.89	−97
5	**2**	MeOH	(*Sa*)/(+)	1.84	−2.72	−7.90	71
6		EtOH	(*Sa*)/(+)	3.54	−2.45	−5.72	156
7		1-PrOH	(*Ra*)/(−)	1.58	1.38	5.53	−24
8		2-PrOH	(*Ra*)/(−)	4.94	3.98	16.52	−32
9	**3**	MeOH	(*P*)/(−)	1.53	−0.52	−0.90	304
10		EtOH	(*P*)/(−)	1.69	−0.55	−0.80	412
11		1-PrOH	(*P*)/(−)	1.46	−0.22	0.02	NA
12		2-PrOH	(*P*)/(−)	1.50	−2.62	−7.97	56
13	**4**	MeOH	(*R*_P_)/(+)	1.55	−0.13	0.43	NA
14		EtOH	(*R*_P_)/(+)	1.75	0.84	3.92	−60
15		1-PrOH	(*R*_P_)/(+)	2.16	0.12	1.95	−209
16		2-PrOH	(*R*_P_)/(+)	2.22	6.51	23.42	5

## Data Availability

Data sharing not applicable.

## Supplementary Materials

The following supporting information can be downloaded at: https://www.mdpi.com/article/10.3390/molecules27238527/s1, Table S1: Effect of mobile phase on the retention of the first eluted enantiomer (*k*_1_) and enantioseparation (*α*) factors of **1**–**4**.

## Sample Availability

Samples of the compounds **1** and **3** can be available from the authors.
