# Supplementary material for: HPLC Enantioseparation of Rigid Chiral Probes with Central, Axial, Helical, and Planar Stereogenicity on an Amylose (3,5-Dimethylphenylcarbamate) Chiral Stationary Phase
Source: Molecules. 2022 Dec 3;27(23):8527. doi: 10.3390/molecules27238527 (PMC9741213; doi:10.3390/molecules27238527)
Supplement: Supplementary file 1 [file molecules-27-08527-s001.zip › molecules-1998325-supplementary.pdf]

# Supplementary Materials

**Table S1**. Effect of mobile phase on the retention of the first eluted enantiomer ($k_1$) and enantioseparation ($\alpha$) factors of **1**-**4**.
Chromatographic conditions: column, Chiralpak AD-3 (100 mm x 4.6 mm, 3 μm); flow rate, 1 mL/min; temperature, 25 °C; detection, UV and CD at 241 nm. 2-PrOH: 2-propanol; 1-PrOH: 1-propanol, EtOH: ethanol; MeOH: methanol.

| *Compound* | *Mobile phase* | $k_1$ | $\alpha$ |
|---|---|---|---|
| **1** | MeOH | 0.77 | 1.23 |
| | EtOH | 2.17 | 2.00 |
| | 1-PrOH | 16.6 | 1.81 |
| | 2-PrOH | 32.74 | 1.47 |
| **2** | MeOH | 1.99 | 1.84 |
| | EtOH | 2.15 | 3.54 |
| | 1-PrOH | 1.55 | 1.58 |
| | 2-PrOH | 2.44 | 4.94 |
| **3** | MeOH | 1.40 | 1.53 |
| | EtOH | 1.44 | 1.69 |
| | 1-PrOH | 1.07 | 1.46 |
| | 2-PrOH | 1.13 | 1.50 |
| **4** | MeOH | 0.33 | 1.55 |
| | EtOH | 0.28 | 1.75 |
| | 1-PrOH | 0.26 | 2.16 |
| | 2-PrOH | 0.44 | 2.22 |
